# Netherton Syndrome: A Comprehensive Literature Review of Pathogenesis, Clinical Manifestations, and Therapeutic Strategies

**DOI:** 10.34763/jmotherandchild.20252901.d-25-00014

**Published:** 2025-09-02

**Authors:** Martyna Mocarska, Adrianna Muciek, Julia Dolinkiewicz, Anna Maria Maryńczak, Nicole Nitschke, Katarzyna Strakowska, Laura Opalska, Anna Maria Orłowska

**Affiliations:** Gabriel Narutowicz Municipal Specialist Hospital, Cracow, Malopolska, Poland; The University Hospital in Krakow, Malopolska, Poland; Independent Community Health Care Centre in Garwolin, Mazowieckie, Poland; Clinical Provincial Hospital No. 2 them. Saint Jadwiga the Queen in Rzeszów, Subcarpathia, Poland; The Ludwik Rydygier Memorial Specialised Hospital, Cracow, Malopolska, Poland

**Keywords:** Atopic dermatitis, Netherton syndrome, trichorrhexis invaginata, bamboo hair, SPINK5 gene

## Abstract

Netherton syndrome (NS) is a rare, autosomal recessive genodermatosis resulting from mutations in the SPINK5 gene, which encodes the LEKTI (Lympho-Epithelial Kazal-type-related inhibitor) protein. This deficiency leads to dysregulated epidermal protease activity, primarily of kallikrein-related peptidases (KLKs), causing severe skin barrier defects, abnormal desquamation, and a complex immune dysregulation involving the T_H_2 and T_H_17 pathways. Clinically, NS is characterised by a triad of ichthyosiform erythroderma (often evolving from congenital ichthyosiform erythroderma to ichthyosis linearis circumflexa); pathognomonic hair shaft abnormalities, such as trichorrhexis invaginata (“bamboo hair”); and atopic manifestations with elevated serum IgE.

Diagnosis can be challenging due to symptomatic overlap with other inflammatory dermatoses, congenital ichthyosis, and primary immunodeficiencies. Confirmation relies on clinical findings, trichoscopic hair examination, and SPINK5 genetic testing. Management is currently largely supportive, focusing on emollients, antiseptics, and cautious use of topical anti-inflammatory agents. While traditional systemic treatments have limitations, emerging targeted therapies, including biologics and gene therapy, show promise, but require further investigation through robust clinical trials to establish their efficacy and safety. This review highlights the diagnostic intricacies and evolving therapeutic landscape of this complex disorder.

## Background

NS is a rare. genetic and potentially life-threatening dermatosis associated with immunodeficiencies. To provide a historical overview, the disease was described first by Comèl in 1949 [1]; later, in 1958, Dr. E. W. Netherton described a bamboo-like deformity in the fine hair of a girl with erythematous-exfoliative dermatitis [2]. The definition of Netherton syndrome evolved with patient observation to include trichorrhexis invaginata, ichthyosis linearis circumflexa, and immune dysregulation [3].

## Epidemiology

Netherton syndrome (NS) is recognised as a rare inherited condition. Its incidence is most commonly cited as approximately one in 200,000 live births [3,4,5]. However, there is reason to believe that this figure might be an underestimation. Some research suggests the actual incidence could be considerably higher, potentially around one per 50,000 births. This discrepancy may arise due to difficulties in accurately diagnosing Netherton syndrome, as its symptoms can overlap significantly with other skin conditions, including atopic dermatitis, various congenital erythrodermas, and certain forms of ichthyosis [4].

Despite the potential underreporting, the overall prevalence of NS (which measures the number of individuals living with the condition at a specific time) is estimated to range from one to nine per 1,000,000 people [5].

NS is a notable cause of congenital erythroderma, which is characterised by widespread skin redness present from birth or shortly after. It is thought that NS may be responsible for as many as 18% of these cases [3].

Although the skin and hair abnormalities associated with Netherton syndrome typically persist throughout a person’s life, the severity of the disorder often lessens with age. Nevertheless, the prognosis can be serious, especially during infancy. Potentially life-threatening complications can arise, contributing to significant mortality in the first few years of life [5].

## Clinical manifestations

NS presents with a characteristic triad of clinical manifestations that typically emerge in early infancy:

### Cutaneous manifestations

The most prominent and early-presenting feature of NS is severe skin involvement, which typically manifests in two distinct patterns: Congenital ichthyosiform erythroderma (CIE), which can persist during childhood or evolve into Ichthyosis Linearis Circumflexa (ILC). CIE presents as generalised erythema and widespread scaling of the skin. Infants with the most severe course of disease may be born with collodion membranes. ILC manifests inconsistently among patients and its prevalence may fluctuate in response to seasonal climate variations [3].

### Hair abnormalities

Trichorrhexis invaginata, otherwise referred to as “bamboo hair,” is a pathognomonic sign of NS. This condition is characterised by patchy thinning of the hair. However, complete alopecia is rare. It is noteworthy that hair growth frequently improves with age, and in some cases, complete resolution has been observed. Additionally, sparse eyebrows, particularly in the lateral sections, are frequently observed. Furthermore, the presence of “golf tee” hairs can be detected as a consequence of the rupture of the hair shaft at the level of invagination, resulting in the formation of a cup-shaped portion of the shaft. Another hair shaft abnormality that can be encountered is the matchstick sign, consisting of a broken hair shaft with a bulging end. These hair shaft defects have been documented in various locations including the scalp, eyebrows, and eyelashes. However, some authors have reported a higher prevalence of trichorrhexis invaginata and “golf tee” hairs in the eyebrows compared to the scalp. Other hair shaft abnormalities reported in NS include pili torti, trichorrhexis nodosa, and helical hairs [3, 6, 7].

### Immune dysregulation

The disease typically manifests through atopic predisposition, accompanied by elevated serum levels of immunoglobulin E (IgE). Immunological alterations, including B-cell immunodeficiency and selective antibody deficiency, have been documented in NS [5]. The presence of eczematous-like lesions, severe pruritus, upper airway infections and multiple food allergies with elevated serum IgE levels is a constant feature. Netherton syndrome is characterised by flare-ups accompanied by the development of lesions that are susceptible to infection [8].

Beyond the primary skin manifestations, hair abnormalities and immune system dysfunction, Netherton syndrome encompasses a diverse range of systemic issues and complications. Infants, particularly those exhibiting more severe symptoms, are predisposed to failure to thrive and hypernatremic dehydration; this is primarily attributable to excessive fluid loss through a compromised skin barrier, in conjunction with impaired thermoregulatory function. Although rare, the development of serious infections such as bronchopneumonia or sepsis in infants is a possibility [3]. Furthermore, individuals may experience chronic non-infectious diarrhoea, short stature, and potential intellectual disability or neurological deficits, alongside a heightened risk of infections. It is particularly important to emphasise that long-term monitoring is crucial due to their increased risk of developing skin cancer later in life [6].

## Genetic basis and pathogenesis

Netherton syndrome is an autosomal recessive genodermatosis caused by loss-of-function mutations within the *SPINK5* (serine protease inhibitor Kazal-type 5) gene. [4, 8,9,10] This gene resides on chromosome 5q31–32 [4, 6, 9] and encodes the multi-domain serine protease inhibitor known as LEKTI (lympho-epithelial Kazal-type-related inhibitor) [4,5,6, 8,9,10]. As of 21^st^ May 2025, 129 distinct pathogenic variants in *SPINK5* associated with NS have been catalogued [11]. Physiologically, LEKTI is expressed within the granular layer of the epidermis, the hair follicles, stratified epithelia of the skin and mucosa, and thymic Hassall’s corpuscles [4,5,6, 9, 10].

**Figure 1.**
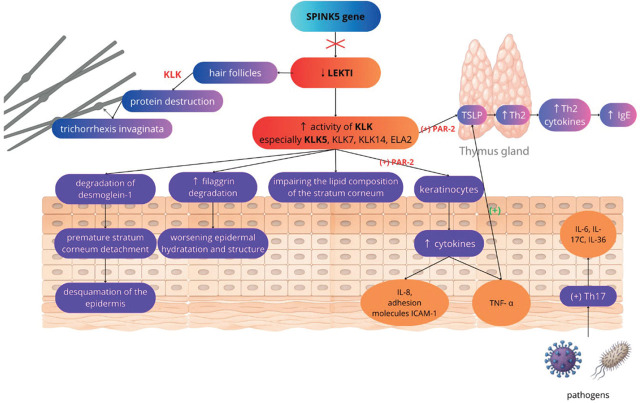
Pathogenesis of NS. LEKTI: lympho-epithelial Kazal-type-related inhibitor, KLK: kallikrein-related peptidases; TSLP: thymic stromal lymphopoietin; ELA2: elastase 2.

The pathogenesis of Netherton syndrome stems directly from the functional loss of LEKTI, as demonstrated by Figure 1 [4, 8, 10]. LEKTI normally functions as an endogenous inhibitor of various serine proteases — including plasmin, trypsin, subtilisin A, cathepsin G and elastase — and has a primary regulatory role in controlling the activity of epidermal kallikrein-related peptidases (KLKs) — notably, KLK5, KLK7, and KLK14 [4, 6, 8, 10]. Under physiological conditions, LEKTI inhibits these KLKs in the lower-stratum corneum, dissociating from its targets in the upper-stratum corneum in a pH-dependent manner and thereby facilitating regulated epidermal desquamation [4, 8].

In NS, LEKTI’s absence or deficiency results in the aberrant, unrestricted activity of these epidermal serine proteases. This is particularly evident for KLK5, which subsequently hyperactivates downstream proteases, including KLK7, KLK14, and elastase 2 (ELA2). This dysregulated proteolytic cascade precipitates profound disturbances in epidermal barrier homeostasis, leading to the core clinical features of NS [4, 10].

Uncontrolled KLK activity profoundly compromises the integrity of the stratum corneum. Elevated KLK5 activity mediates the degradation of key structural proteins, such as the corneodesmosomal component desmoglein 1, resulting in premature stratum corneum detachment and NS’s characteristic excessive scaling (desquamation) [6, 10]. KLK hyperactivity also increases degradation of filaggrin, a protein that plays a crucial role in epidermal hydration and structure, while also impairing the lipid composition of the stratum corneum; these are both essential for barrier competence [8, 10]. Collectively, these events culminate in a severely defective epidermal barrier with abnormal homeostasis, increased transepidermal water loss, and heightened vulnerability to environmental insults, including the penetration of microbes and allergens [4, 6, 8, 10,12].

The consequences of LEKTI deficiency extend significantly into immune activation and inflammation. KLK5, through the activation of PAR-2 on keratinocytes, triggers the production and release of multiple pro-inflammatory and pro-allergic mediators. Among the most significant are cytokines, including IL-8, TNF-α, and the adhesion molecule ICAM-1, which collectively promote inflammatory processes [5, 6, 10].

Concurrently, the KLK5-PAR2 axis strongly induces the expression of Thymic Stromal Lymphopoietin (TSLP) [5, 6, 10]. TSLP is a potent cytokine known to drive T helper type 2 (T_H_2) immune responses. Its overexpression in NS promotes T_H_2 cell differentiation, leading to increased production of T_H_2 cytokines and elevated levels of serum Immunoglobulin E (IgE), and this fosters atopic diathesis, including eczema-like lesions, allergies, and asthma [6, 8, 10]. Furthermore, TNF-α and IL-1 increase the secretion of TSLP, which acts in synergy with these pro-inflammatory molecules, amplifying the expression of pro-T_H_2 cytokines by activated mast cells [5].

In parallel, the chronically-impaired skin barrier facilitates the entry of pathogens and danger signals. This is thought to drive a T helper type 17 (T_H_17) response, characterised by the production of cytokines such as IL-6, IL-17C and IL-36. Therefore, the immunological landscape of NS is complex and shares features with T_H_2-driven atopic dermatitis and T_H_17-driven psoriasis [8].

### Thymic involvement and systemic immunity

Beyond the skin, LEKTI is also expressed within the Hassall’s corpuscles of the thymus. These are structures involved in thymic function, including the potential induction of regulatory T cells. Given the thymus’s central role in T-cell development, defective LEKTI expression in this organ has led to hypotheses that NS might involve an intrinsic systemic immunodeficiency that contributes to the observed increased susceptibility to infections [10].

However, the precise impact of thymic LEKTI deficiency on T-cell maturation and overall systemic immunity remains less well-defined and subject to ongoing investigation. Some studies have reported specific immune defects in subsets of NS patients, such as decreased numbers and functional immaturity of Natural Killer (NK) cells; reduced memory B cell populations; and impaired responses to polysaccharide vaccines. However, compelling evidence for a severe, clinically relevant primary systemic immunodeficiency as a universal feature of NS is lacking in some larger cohort studies. Detailed immunophenotyping in certain cohorts did not reveal significant abnormalities in major lymphocyte subsets or immunoglobulin levels typically associated with primary immunodeficiencies, although subtle alterations, like lower T_H_1 cell counts, were noted. These findings suggest that while subtle immune alterations may exist, the profound skin-barrier defects could be the principal driver for the recurrent infections and allergic sensitisation that are characteristic of NS. The rarity of the disease and historical reliance on small, often paediatric cohorts have limited definitive conclusions regarding long-term systemic immune consequences [10].

## Diagnosis

The diagnosis of Netherton syndrome is based on a comprehensive evaluation of the clinical presentation and a thorough examination of hair structure, along with a range of laboratory tests and genetic testing. For a diagnosis to be made, three characteristics are typically required: ichthyosiform erythroderma, a specific hair shaft abnormality known as trichorrhexis invaginata, and high serum IgE levels with atopic manifestations [13]. In addition, laboratory findings may show peripheral eosinophilia [9]. Genetic testing should be considered when the clinical picture resembles Netherton syndrome. This is necessary to confirm the diagnosis by identifying pathogenic variants in the *SPINK5* gene [6].

**Figure 2.**
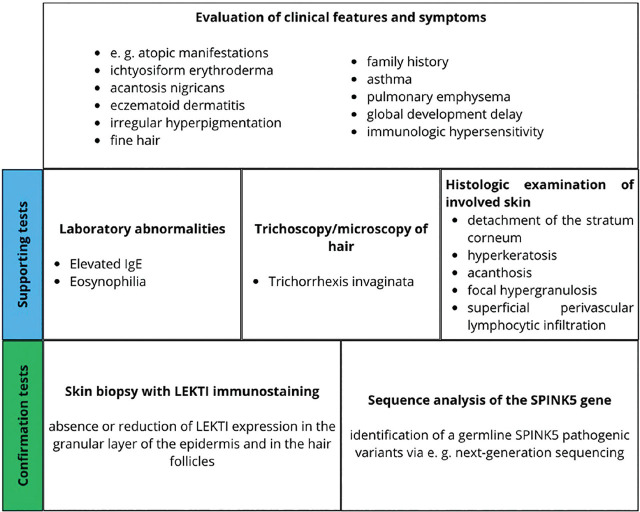
Diagnosis of Netherton syndrome.

Figure 2 presents a concise synopsis of the diagnostic capabilities. Prenatal diagnosis of NS has now become a viable option, with the disease-causing *SPINK5* mutations of the family becoming identifiable as early as the 10th to 12th gestational week [7].

## Differential diagnosis

The differential diagnosis of Netherton syndrome encompasses three principal categories of disorders (Table 1). First and foremost are inflammatory dermatoses like atopic dermatitis, seborrhetic dermatitis, and acrodermatitis enteropathica [14], which often have similar cutaneous manifestations to NS [6, 15, 16]. The second major group comprises congenital ichthyosis [6], which share phenotypic features like generalised scaling and skin barrier dysfunction, though typically without the immunological or hair anomalies characteristic of NS.

**Table 1.** Differential diagnosis of Netherton syndrome.

**Category**	**Representative Conditions**	**Overlapping Features with NS**	**Distinguishing Features**
Inflammatory Dermatoses	Atopic dermatitis, deborrheic dermatitis, acrodermatitis enteropathica	Erythema, pruritus, eczematous or scaling lesions, onset in infancy	No trichorrhexis invaginata; transient course; response to topical therapy or zinc
Congenital Ichthyoses	ARCI, Ichthyosis vulgaris	Generalised scaling, skin barrier dysfunction	Absence of atopy and bamboo hair
Primary Immunodeficiency Disorders (PIDDs)	Hyper-IgE syndrome, (STAT3/DOCK8 deficicency); IPEX, Omenn, or Wiskott-Aldrich syndrome	Elevated IgE, eosinophilia, recurrent infections, eczema-like dermatitis	No LEKTI deficiency; systemic autoimmunity; hematologic anomalies (thrombocytopenia)

ARCI: autosomal recessive congenital ichthyosis; IPEX: immune dysregulation polyendocrinopathy enteropathy X-linked syndrome; LEKTI: lympho-epithelial Kazal-type-related inhibitor.

A third and diagnostically critical category includes primary immunodeficiency disorders (PIDDs). While these conditions may not closely resemble NS in terms of cutaneous morphology, they often present with systemic atopic features and laboratory abnormalities that overlap with NS, such as markedly elevated serum IgE levels, eosinophilia, and recurrent infections [10, 14, 15]. PIDDs to consider in this context include hyper-IgE syndrome (STAT3 or DOCK8 deficiency) [15], immune dysregulation, polyendocrinopathy, enteropathy, X-linked syndrome (IPEX syndrome), Omenn syndrome [14, 17], Wiskott-Aldrich syndrome [14, 15], and other rare combined immunodeficiencies. Careful clinical evaluation, trichoscopic analysis for trichorrhexis invaginata [6, 16], immunohistochemical staining for LEKTI [6], and targeted genetic testing remain pivotal in distinguishing Netherton syndrome from these similar conditions [7].

## Treatment

Given the complex pathogenesis of the disease, no targeted therapies are currently available for patients with NS. Early diagnosis is essential to initiate appropriate management. Optimal care requires a multidisciplinary team involving specialists in immunology, allergology, and dermatology. The most suitable therapeutic strategy should be determined based on the individual clinical features of each patient affected by this rare condition [5].

The first line of treatment for congenital ichthyosis is local therapy. To prevent recurrent skin infections, antiseptics, such as chlorhexidine, potassium permanganate, polyhexanide and octenidine, are recommended for use two to three times a week. Emollients are also effective in patients who have a damaged skin barrier, moisturizing and lubricating the skin while also forming an occlusive layer. Using them several times a day is recommended, especially immediately after bathing [6].

Topical corticosteroids, which are frequently employed as a fundamental component in the management of numerous dermatoses due to their anti-inflammatory and antiproliferative properties, should be utilised with caution in cases of NS. Their use should be restricted to low-potency formulations, applied for short durations and limited to small, actively affected areas of the body [5]. Topical calcineurin inhibitors are likewise advised for short-term use and should be applied to localised areas during disease flares [18].

Additionally, calcipotriol, a synthetic vitamin D analogue with antiproliferative properties that promotes terminal epidermal differentiation, may be utilised as a topical treatment. This approach is beneficial in NS, where keratinocyte proliferation is enhanced and differentiation is impaired [5, 19]. Selection and appropriate duration of topical treatments in NS are both important, considering the increased absorption secondary to the skin barrier defect [6].

Other therapies include phototherapy, including narrow-band UVB, psoralen plus UVA (PUVA), or UVA-1, which has been reported as having short-term efficacy in the majority of case studies. Nevertheless, phototherapy, including UVB, is not recommended, even for short-term management, and its long-term use is particularly discouraged due to the significant risk of skin cancer [18].

Systemic immunoglobulins have demonstrated safety and efficacy in a small number of patients. However, their use cannot be routinely recommended due to limited supporting evidence and a lack of long-term data [18].

Recent advancements in understanding the pathophysiology of NS have facilitated the development of more targeted biological therapies. Due to increased Il-17A signalling, four patients were treated with secukinumab, achieving reduced itching with topical steroid use. Furthermore, both pediatric patients showed improved growth velocity at the six-month assessment [20]. IL-17 inhibition appears to be the most effective treatment; however, evidence is limited to case reports involving small patient cohorts and lacks placebo-controlled data [8].

Another reported therapeutic strategy is blocking the p40 subunit of interleukin-12 and interleukin-23 with ustekinumab. A case report documented significant clinical improvement with no relapse observed after one year of administering treatment every three months [21]. Omalizumab, a humanised monoclonal antibody that selectively targets IgE, was also reported to reduce allergic skin symptoms in a 20-year-old male patient with NS [22].

Several studies have suggested dupilumab as a potential treatment for NS, demonstrating improvements in clinical signs and symptoms, such as pruritus and scaling, in both adult and paediatric patients [23,24,25]. Dupilumab targets the interleukin-4 receptor alpha subunit, which is a shared component of the IL-4 and IL-13 receptor complexes. Consequently, it blocks the signalling pathways mediated by these cytokines, which play a central role in the T_H_2-lymphocyte-driven immune response associated with NS [8].

Treatment with infliximab, a TNF-α inhibitor, is not recommended because an increased risk of skin cancer and recurrent infections was reported in patients [5, 6]. The latest guidelines for the management of congenital ichthyosis recommend the use of biologic drugs chiefly in the severe erythrodermic form of congenital ichthyosis [26].

Several ongoing studies are exploring novel therapeutic targets for the treatment of NS. Given the elevated activity of KLK5 observed in Netherton syndrome, multiple targeted KLK5 inhibitors are currently being developed [27, 28]. Di et al., in their Phase 1 trial, confirmed the safety and feasibility of gene therapy involving autologous keratinocytes transduced with a lentiviral vector encoding *SPINK5* [29, 30]. Although still in the early stages of development, this approach shows promise as a potential treatment option.

## Conclusions

Netherton syndrome represents a rare, autosomal recessive genodermatosis characterised by a triad of clinical features — ichthyosiform erythroderma, trichorrhexis invaginata (“bamboo hair”) [3], and marked atopy with elevated IgE levels [15]. This article provides a comprehensive overview of current diagnostic criteria, recent genetic discoveries, differential diagnoses, and evolving treatment options, reflecting the complexity and heterogeneity of this disorder.

Diagnosis of NS is a significant clinical challenge due to a variety of symptoms and similarities to many inflammatory skin diseases [14]. As detailed, clinical suspicion must be supported by histological and trichoscopic examination of involved skin and hair [9, 16] and laboratory tests for elevated IgE levels and eosinophilia [9]. Genetic testing plays a crucial role in confirming the diagnosis of NS by detecting pathogenic variants in the *SPINK5* gene [6].

The core pathogenesis of NS involves epidermal protease hyperactivity due to LEKTI deficiency, leading to severe skin barrier disruption and a complex cutaneous inflammatory milieu characterised by mixed T_H_2 and T_H_17 pathway activation [8]. While LEKTI’s role in the thymus raises questions about potential effects on systemic immunity, the extent to which this contributes to the clinical phenotype — distinct from the severe consequences of the barrier defect — requires further elucidation [10].

Management of NS remains largely supportive and symptomatic, reflecting the lack of disease-specific therapies. Early diagnosis is crucial for the implementation of appropriate therapeutic interventions, as it can prevent serious or even fatal complications, such as dehydration, secondary skin and respiratory infections, and hypothermia [9].

The primary treatment for Netherton syndrome involves local therapies, including regular use of antiseptics to prevent secondary infections and frequent application of emollients to restore the skin barrier [6]. Topical corticosteroids and calcineurin inhibitors may be used with caution during flares [5], while agents like calcipotriol help regulate keratinocyte proliferation [18]. Due to the risk of adverse effects, phototherapy and systemic immunoglobulins are not routinely recommended [18]. Recent advances in targeted biologics, such as IL-17 inhibitors (secukinumab) [20], IL-12/23 blockers (ustekinumab) [21], anti-IgE therapy (omalizumab) [22], and IL-4/IL-13 inhibitors (dupilumab) [23,24,25], show promise in managing symptoms, though the evidence remains limited to case reports. TNF-α inhibitors like infliximab are discouraged due to their associated risks [5, 6]. Biologic therapies are mainly reserved for severe erythrodermic cases, per current guidelines.

In conclusion, this article provides a detailed and up-to-date overview of NS, articulating both the diagnostic intricacies and therapeutic challenges. While first-line treatment of NS is still symptomatic, new findings offer hope for improved outcomes. Further research — particularly randomised controlled trials — will be essential to understanding the pathogenesis of the disease and systematising promising research results related to biological treatment.

### Key points

- Netherton syndrome is a rare, autosomal recessive disorder caused by *SPINK5* gene mutations, leading to LEKTI protein deficiency.
- It is clinically characterised by the triad of ichthyosiform erythroderma, trichorrhexis invaginata (“bamboo hair”), and atopic manifestations with high serum IgE.
- Diagnosis relies on clinical findings, hair analysis, and *SPINK5* genetic testing.
- Management is primarily supportive and includes emollients and antiseptics, with cautious use of topical anti-inflammatory agents.
- Emerging targeted therapies, such as biologics, show promise, but require more extensive clinical trials.
- Differential diagnosis is broad, encompassing other inflammatory dermatoses, congenital ichthyoses, and primary immunodeficiency disorders.
